# A study on the effects of health behavior and sports participation on female college students' body mass index and healthy promoting lifestyle

**DOI:** 10.3389/fpubh.2022.1069219

**Published:** 2023-01-04

**Authors:** Xiangyun Lin, Hao Liu

**Affiliations:** ^1^Department of Physical Education, North Sichuan Medical College, Nanchong, China; ^2^College of Physical Education, Southwest Medical University, Luzhou, China

**Keywords:** demographic variables, socio economic background, body shape, health promoting lifestyle, abdominal obesity

## Abstract

**Objective:**

Life form and body composition may affect the health of college students. This study will explore the relationship between the demographic variables of female college students and their body composition and health promoting lifestyle, so as to provide useful reference for the future design and planning of college students' physical and mental health courses and improving their physical activity level.

**Methods:**

Using the method of questionnaire and bioelectrical resistance measurement, a questionnaire on healthy lifestyle of college students was constructed on the basis of consulting a large number of relevant research literature. Relevant survey samples were obtained through random sampling, and their body composition was measured; use SPSS21.0 statistical analysis software to conduct statistical analysis on relevant indicators.

**Results and conclusions:**

(1) at present, female college students generally lack leisure activities and sleep, the proportion of regular fitness habits is low, and the number of snacks and average daily online time are generally too high; The overweight rate and body fat rate of female college students are generally too high, while the standard rate of muscle weight is generally too low. (2) Female college students' health promoting lifestyle has the highest score of self-realization, followed by interpersonal support and the worst behavior of sports participation; The older the college students, the worse their sports participation and overall health promotion behavior; The better the Conscious health status, the better the self-realization, exercise participation and nutritional behavior; The more exercise time per day, the higher their participation in sports and the stronger their health responsibility; The more time spent on the Internet every day, the worse the health responsibility and sports participation. (3) The more time female college students spend on the Internet every day, the higher the probability of overweight. Those with regular exercise habits have a lower proportion of overweight and high body fat rate, while the better their sleep and night snack behavior, the lower their body fat rate. The older college students are, the larger their visceral fat area is, the better their exercise habits and sleep behavior are, the smaller their visceral fat area is, and the lower their visceral fat level is.

## 1. Introduction

Modern technology and network facilitation have changed the eating habits of modern people, increased the pressure of life, reduced leisure time and physical activity time, resulted in the deterioration of physical activity and lack of exercise, thereby affecting health, increasing the risk of chronic diseases (such as hypertension, diabetes, etc.) and degenerative diseases of body function (such as back pain, joint lesions, etc.) (1–3). The outline of healthy the China 2030 plan is a strategic deployment formulated to promote the construction of a healthy China and improve people's health. The outline pays more attention to the active promotion rather than negative maintenance of people's health (4). Contemporary college students should shoulder the important task of building socialism with Chinese characteristics, but according to relevant domestic research reports, the score of overall health promoting lifestyle of college students in China is only limited to the medium level, while the performance of health responsibility and sports behavior is very poor (5–8). In relevant foreign studies, the lifestyle of college students also has the phenomenon of insufficient physical activity, but the intervention of health promoting lifestyle (such as diet and health education, exercise, improving static behavior, etc.) can effectively control and improve the physique, BMI, cortical thickness and hip circumference of obese adolescents (9, 10). The compound intervention of healthy eating habits and sports education program can significantly reduce the incidence of BMI and obesity in 62% of school children (11). The health promotion exercise program has significant positive effects on improving the risk factors (triglycerides, total cholesterol, glycosylated hemoglobin, etc.) of high-risk groups of metabolic diseases, and can promote the formation of regular exercise habits (12, 13).

Body composition refers to the percentage of body adipose tissue and non-adipose tissue (bone and muscle) in body weight, which is often used as the basis for evaluating health, exercise ability and healthy physical fitness (14). The body composition of adolescents (especially college students) is affected by gender. Among them, boys are significantly higher than girls in muscle mass, waist hip ratio and visceral fat, while the amount and percentage of body fat are significantly lower than girls (15). At the same time, different departments, schoolwork pressure and participation in leisure activities will also affect body composition (16). Studies have shown that high body fat, insufficient muscle mass and physical weakness can induce the risk of juvenile sarcopenia and obesity (17). Obesity can be caused by excessive body fat and obesity can cause comorbidify such as diabetes, hypertension, hyperlipidemia and mental disorders (such as social impairment and depression) (18). The 4 years of college life is an important and critical period for teenagers to enter adulthood. If they can cultivate their good and healthy lifestyle in this period, they will greatly reduce their risk of chronic diseases in the future. However, according to the latest domestic research literature, there are few related studies on college students' health promotion lifestyle and body composition. This study combines cross-sectional survey and body composition measurement to reveal the relationship between demographic variables, health promoting lifestyle and body composition of college students in China, so as to provide a useful reference for designing and planning college students' physical and mental health courses and improving their physical activity levels in the future.

## 2. Research object and method

### 2.1. Research object

This study adopts a cross-sectional study design. Taking the freshman girls of a comprehensive university in the central region as the survey and test objects, 100 people were selected from 12 disciplines such as philosophy, economics, law, education, literature, history, science, engineering, agriculture, medicine, management and art, and a total of 1,200 samples were obtained. Taking the opportunity of physical fitness test for freshmen in colleges and universities, questionnaires and corresponding physical fitness monitoring were conducted for these subjects from October 2021 to November 2021.

### 2.2. Data collections and research ethics

This study was approved by the human trial ethics committee. Before the questionnaire survey, the researcher explained the purpose and method of the study to the participants, and the data would not to be used for purposes other than the study. The questionnaire was issued after obtaining the informed consent of the subjects, and the physical composition test was arranged.

### 2.3. Research methods

#### 2.3.1. Questionnaire survey method

The questionnaire structure consists of three parts:

- Personal basic data include gender, age, health status, working hours, regular participation in sport or leisure community activities, regular exercise habits, sleep habits, Internet hours and night snack habits.
- Health Promoting Lifestyle scale for college students. The “adolescent health promoting lifestyle scale” of Chen et al. (19) was modified to evaluate the performance of health promoting lifestyle of participants in this study (see Table 1).

**Table 1:** Common factor extraction and reliability analysis of college students' health promoting lifestyle scale.

**KMO Bartlett spherical test**	**Factor naming**	**Number of items**	**Eigenvalue**	**Explained variation%**	**Progressive variation%**	**Cronbach α coefficient**
KMO = 0.77						
*P* = 0.000	Nutritional behavior	5	9.15	20.76	20.76	0.73
	Health responsibility	8	7.24	16.43	37.19	0.76
	Self-realization	8	5.62	12.76	49.95	0.75
	Interpersonal support	6	3.71	8.42	58.37	0.81
	Sports participation	4	2.58	5.86	64.23	0.79
	Stress response	9	1.48	3.36	67.59	0.77

Nutritional behavior (5 items): A1, eating breakfast; A2. The time and quantity of three meals per day is normal; A3. The diet contains fiber rich food; A4. Drinking at least 1500 cc of water every day; A5. Daily meals include five categories of food. Health responsibility (8 items): A6. Check cholesterol and know the results; A7. Take blood pressure and know the results; A8. Discuss health-related issues with medical staff; A9. Observe whether your body has changed or abnormal; A10. Participation in education course on personal health knowledge; A11. Do not eat food containing preservatives or artificial additives; A12. Take your pulse when doing exercise; A13. Looking at the nutritional composition of the food. Self realization (8 items): A14. Appreciate yourself; A15. Full of confidence and optimism in life; A16. Growth and change in a positive direction; A17. Know my own advantages and disadvantages; What is important in life; A19. Value your achievements; A20. Every day is full of fun and challenges; A21. Feeling that your life is meaningful. Social support (6 items): A22. Be willing to keep in touching with closing people; A23. Maintain use interpersonal relationships; A24. Staying with closing friends; A25. Showing concern, love and warmth to others; A26. Keeping in touching with the people I care about; A27. Discuss personal issues and concerns. Exercise behavior (4 items): A28. Do stretching exercises at least 3 times / week; A29, 3 times / week, 20-30 minutes of exercise each time; A30. Participate in sport courses or activities guided by others; A31. Participate in recreational sports. Stress management (9 items): A32. Find some time to relax every day; A33. Practice relaxation for 15-20 minutes every day; A34. Know the source of stress in life; A35. Pay attention to control your weight; A36 will pay attention to their unpleasant emotions; A37. Sleep 6 to 8 hours a day; A38. Arrange work and rest time in a planned way; A39. My response to unreasonable requests is appropriate; A40. Reading health promoting newspapers, magazines or books.

This scale contains 40 questions and can extract six common factors (KMO = 0.77 and Bartlett spherical test value is significant (*P* < 0.001), and the cumulative explained variation of six common factors is 67.59%. The first common factor contains five items, and its contribution rate is 20.76%. The content is related to teenagers' daily diet and food choice, so it is named “nutritional behavior” factor; The second common factor contains eight items, with a contribution rate of 16.43%, which mainly involves teenagers' attention to health, such as routine physical examination, self-health topic discussion, health education and training and daily food choice, so it is named “health responsibility” factor; The third public factor contains eight items, with a contribution rate of 12.76%, which involves teenagers' cherishing of life, growth mentality, knowing their strengths and weaknesses, and how to face achievements and challenges, so it is named “self realization” factor; The fourth common factor contains six items, with a contribution rate of 8.42%. The content mainly involves the interpersonal relationships that teenagers have and the related support they can get from them, so it is named “interpersonal support” factor. The fifth common factor contains four items, with a contribution rate of 5.86%, which mainly involves teenagers' sports and fitness problems, such as exercise time, intensity and frequency, so it is named “sports participation” factor; The sixth common factor contains nine items, with a contribution rate of 3.36%, which mainly involves how teenagers face pressure, mitigation methods and countermeasures, so it is named “stress response” factor. The internal consistency test results of six common factors show that Cronbach's α the coefficients are 0.73, 0.76, 0.75, 0.81, 0.79 and 0.77 respectively, indicating that the adolescent health promotion lifestyle scale has good measurement validity (see Table 1).

#### 2.3.2. Measurement method

The body composition analyzer (IOI353; origin: Korea) was used to measure the body composition of participants, including body weight, body mass index, body fat weight, body fat percentage, muscle weight, visceral fat degree, visceral fat area, abdominal obesity rate, waist circumference ratio, visceral fat weight and subcutaneous fat weight.

#### 2.3.3. Mathematical statistics

SPSS 21.0 statistical software package was used for data processing, and descriptive statistics were used to explore the subjects' basic attributes, health promoting lifestyle, body composition and abdominal obesity indicators; Independent sample *t*-test, chi square test, one-way ANOVA and Pearson correlation analysis were used for inferential statistical analysis. The significance level of all indicators is set as α= 0.05.

## 3. Results

### 3.1 Descriptive analyses of demographic variables of college students

Table 2 shows:

- among 1,200 subjects, the average age is 21.69±2.61 years. In “Compared with people of the same age, I think my health status is...”, the score is 6.91 ± 1.78; 42.8% of them have fixed leisure community activities, Regular exercise habits accounted for 34.2%, 55.0% were lack of sleep, The average daily online time is about 5.58 ± 3.75 h, Only 43.6% of them had no habit of eating snacks.
- From the physical state of the subjects, the average weight of the students was 56.59 ± 8.52 kg, the overweight rate was 21.4%, the lean accounted for 24.7%, and the weight standard accounted for 53.9%. The mean value of body mass index (BMI) is 22.35 ± 3.08 kg/m^2^, of which 28.6% is too high, 13.6% are too low and 57.8% is standard. The average body fat weight was 14.55 ± 5.32 kg and the average percentage of body fat was 25.23 ± 5.17%. The percentage of body fat that was too high accounted for 18.3%, the percentage of body fat that was too low accounted for 10.3%, and the standard accounted for 71.4%. The average muscle weight was 36.06 ± 3.87 kg, 13.6% were too high, 27.5% were insufficient and 58.9% were standard.
- From the abdominal condition of the subjects, the average area of visceral fat was about 11.09 ± 5.47 cm^2^, with visceral obesity accounting for 15.0%, subcutaneous type accounting for 60.8%, and balanced type accounting for only 24.2%. The mean value of visceral fat was 5.22 ± 2.84 kg. Based on this, it was judged that visceral obesity accounted for 19.2%, subcutaneous type accounted for 56.6%, and balanced type only accounted for 34.2%. The average abdominal obesity rate (waist circumference ratio) was 0.76 ± 0.12. According to this, 11.7% were judged to be too high, 9.4% were lower, and 78.9% were judged to be standard. The mean weight of visceral fat was 1.58 ± 0.69 kg, and the mean weight of subcutaneous fat was 12.97 ± 4.09 kg.

**Table 2:** Statistical table of basic information and physical condition of respondents (*N* = 1,200).

**Variable name**	**Frequency**	**%**	**Variable name**	**Frequency**	**%**	**Variable name**	**Frequency**	**%**
**Weight (KG)**			**Muscle weight (KG)**			**Waist circumference ratio**		
Thin	296	24.7	Low	330	27.5	Low	113	9.4
Standard	647	53.9	Standard	707	58.9	Standard	947	78.9
Overweight	257	21.4	Overtop	163	13.6	Overtop	140	11.7
Mean value	56.59 ± 8.52		Mean value	36.06 ± 3.87		Mean value	0.76 ± 0.12	
**Body mass index**			**Visceral fat area(cm** ^**2**^ **)**			**Fixed leisure activities**		
Low	163	13.6	Subcutaneous type	730	60.8	None	686	57.2
Standard	695	57.8	Balanced type	290	24.2	Have	514	42.8
Overtop	342	28.6	Visceral obesity	180	15.0	**Exercise habits**		
Mean value	22.35 ± 3.08		Mean value	11.09 ± 5.47		None	790	65.8
**Body fat %**			**Visceral fat level (Kg)**			Have	410	34.2
Low	124	10.3	Subcutaneous type	679	56.6			
Standard	857	71.4	Balanced type	410	34.2			
Overtop	219	18.3	Visceral obesity	111	19.2	Age	21.69 ± 2.61	
Mean value	25.23 ± 5.17		Mean value	5.22 ± 2.84		Body fat weight (kg)	14.55 ± 5.32	
**Night snack habit**			**Sleep quality**			Visceral fat weight (kg)	1.58 ± 0.69	
None	523	43.6	Enough sleep	514	42.8	Subcutaneous fat weight (kg)	12.97 ± 4.09	
1–3 time/week	536	44.7	Sleep debt	660	55.0	Conscious health status	6.91 ± 1.78	
≥4 time/week	141	11.7	Often insomnia	260	2.2	Online time / day (H)	5.58 ± 3.75	

### 3.2. Influence of demographic variables on health promoting lifestyle of female college students

Table 3 data display:

- The average score of female college students' overall health promoting lifestyle was 3.72 ± 0.55, among which the score of self realization was the highest (4.09 ± 0.58), followed by interpersonal support (4.02 ± 0.68), stress coping (3.81 ± 0.73), health responsibility (3.61 ± 0.74), nutritional behavior (3.59 ± 0.81) and sports participation (3.24 ± 0.89).
- From the factors affecting the health promotion lifestyle of female college students, their age is negatively correlated with sport participation (−0.25^*^) and the total score of health promotion (-0.15^*^). The older the students are, the worse their sports participation and overall health promotion behavior are: The conscious health status of female college students is significantly positively correlated with self realization (0.23^*^), exercise participation (0.27^**^), nutritional behavior (0.27^**^) and the total score of health promotion (0.21^*^). Similarly, daily exercise time of female college students is positively correlated with health responsibility (0.21^*^), exercise participation (0.20^*^) and the total score of health promotion (0.19^*^). It means that more female college students exercise every day, the higher their degree of exercise participation and the stronger their health responsibility, the more positive the overall health promotion behavior. In addition, female college students' daily online time is negatively correlated with health responsibility (−0.18^*^) and sports participation (-0.17^*^), which means that the more female college students spend online time every day, the worse their health responsibility and sports participation.
- “Whether there are fixed leisure community activities” has a positive impact on the scores of interpersonal support (*t* = −2.81, *P* < 0.05), sports participation (*t* = −3.70, *P* < 0.05) and health promotion (*t* = −2.16, *P* < 0.05). Female college students “regular exercise habits” positively affect their sports participation scores (*t*= −5.29, *P* < 0.01), nutritional behavior scores (*t* = −3.63, *P* < 0.05) and total health promotion scores (*t* = −2.68, *P* < 0.05). Scores of “regular exercise habits” is higher.
- Sleep behavior and snack behavior of female college students had no significant effect on the six dimensions of health promotion behavior and the total score of health promotion behavior (the corresponding F value was between 0.37 and 2.43, *P* > 0.05).

**Table 3:** Statistical table of the influence of College Students' demographic variables on their health promoting lifestyle.

		**Self**		**Health**		**Interpersonal**		**Sports**		**Stress**		**Nutritional**		**Total score of**	
		**realization**		**responsibility**		**support**		**participation**		**response**		**behavior**		**health promotion**	
		**Mean**	**t/F/r**	**Mean**	**t/F/r**	**Mean**	**t/F/r**	**Mean**	**t/F/r**	**Mean**	**t/F/r**	**MEAN**	**t/F/r**	**Mean**	**t/F/r**
Age (years)		4.09	−0.12	3.61	−0.08	4.02	−0.12	3.24	**−0.25** ^*^	3.81	−0.05	3.59	−0.04	3.72	**−0.15** ^*^
Conscious health status		4.09	**0.23** ^*^	3.61	−0.07	4.02	0.13	3.24	**0.27** ^**^	3.81	0.09	3.59	**0.27** ^**^	3.72	**0.21** ^*^
Exercise time/day		4.09	−0.12	3.61	**0.21** ^*^	4.02	−0.10	3.24	**0.20** ^*^	3.81	−0.07	3.59	−0.05	3.72	**0.19** ^*^
Online time/day		4.09	−0.04	3.61	**−0.18** ^*^	4.02	0.06	3.24	**−0.17** ^*^	3.81	0.08	3.59	0.06	3.72	−0.09
Leisure participation		4.12	−1.39	3.72	−0.51	4.23	**−2.81** ^*^	3.45	**−3.70** ^*^	3.84	−1.40	3.62	−1.42	3.85	**−2.16** ^*^
Regular exercise habits		4.11	−1.26	3.66	−0.29	4.07	−1.29	3.51	**−5.29** ^*^	3.90	−1.66	3.674	**−3.63** ^*^	3.89	**−2.68** ^*^
Sleep	Enough sleep	4.12	0.37	3.55	1.49	3.90	2.43	3.32	1.88	3.83	1.61	3.63	1.82	3.79	1.63
	Sleep debt	4.16		3.61		4.07		3.27		3.81		3.60		3.72	
	Often insomnia	3.98		3.67		4.10		3.15		3.80		3.55		3.65	
Night snack	None	3.98	0.39	3.54	0.47	3.88	2.09	3.30	1.48	3.77	0.99	3.53	1.26	3.68	0.57
	1–3 time/week	4.11		3.57		4.12		3.24		3.82		3.61		3.70	
	≥4 time/week	4.17		3.41		4.06		3.19		3.90		3.64		3.77	
Mean	4.09 ± 0.58		3.61 ± 0.74		4.02 ± 0.68		3.24 ± 0.89		3.81 ± 0.73		3.59 ± 0.81		3.72 ± 0.55	

^*^P < 0.05,

^**^P < 0.01. Among the influencing factors, the relationship between age, Conscious health status, daily exercise time and daily online time on health promotion behavior was analyzed by correlation analysis (the statistic is the correlation coefficient r); The effects of leisure community participation and regular exercise habits on health promotion behavior were tested by independent sample t–test (the statistic was t); The effects of sleep habits and night snack habits on health promotion behavior were analyzed by one–way ANOVA (the statistic is F value)?

### 3.3. Influence of demographic variables on body shape (body weight, BMI, body fat weight, muscle mass) of college students

Table 4 data display:

- Female college students' age, Conscious health status and daily exercise time had no significant effects on their body weight, body mass index (BMI), percentage of body fat (%) and muscle mass; However, the time spent on the Internet every day significantly affected their weight (F = 4.72, P = 0.033 < 0.05), which showed that the people with high weight spent the most time on the Internet every day.
- Whether female college students participate in fixed leisure community activities has nothing to do with their physical state, but whether they have regular exercise habits has a significant impact on their body weight (x^2^ = 5.89, *P* = 0 .024 < 0.05) and body fat percentage (%) (x^2^ = 5.01, *P* = 0.037 < 0.05), which shows that the rate of overweight and excessive body fat percentage of regular exercise habits is lower; The sleep behavior and night snack behavior of college students also affect their body fat percentage. More sufficient sleep (x^2^ = 7.12, P=0.014 < 0.05) and the fewer night snack time (x^2^ = 6.45, *P* = 0.034 . < 0.05), the lower the rate of excessive body fat percentage.

**Table 4:** Statistical table of the influence of College Students' demographic variables on their body shape.

		**Weight (KG)**			**BMI**			**Body fat weight (KG)**			**Body fat (%)**			**Muscle weight (KG)**		
		**In** **sufficient**	**Standard**	**Over** **weight**	**In** **sufficient**	**Standard**	**Over** **weight**	**In** **sufficient**	**Standard**	**over** **weight**	**In** **sufficient**	**Standard**	**over** **weight**	**In** **sufficient**	**Standard**	**Over** **weight**
		**M**	**X** ^2^ **/F**	**P**	**M**	**X** ^2^ **/F**	**P**	**M**	**X** ^2^ **/F**	**P**	**M**	**X** ^2^ **/F**	**P**	**M**	**X** ^2^ **/F**	**P**
Age (years)		56.59	0.84	0.57	22.35	0.57	0.56	14.55	0.73	0.64	25.23	0.58	0.64	36.06	1.26	0.37
Conscious health status		56.59	1.56	0.39	22.35	1.34	0.33	14.55	−0.16	0.51	25.23	1.49	0.26	36.06	0.75	0.48
Exercise time/day		56.59	1.89	0.15	22.35	0.76	0.48	14.55	−0.11	0.77	25.23	0.22	0.62	36.06	0.61	0.54
Online time/day		56.59	**4.72** ^*^	**0.033**	22.35	0.46	0.71	14.55	−0.18	0.50	25.23	0.17	0.81	36.06	1.25	0.23
Leisure participation		56.01	1.49	0.36	21.82	1.65	−0.53	14.63			25.69	0.28	0.75	36.97	1.87	0.33
Regular exercise habits		54.74	**5.89** ^*^	**0.024**	22.39	1.67	0.94	15.57			26.51	**5.01** ^*^	**0.037**	37.31	0.86	0.79
Sleep	Enough sleep	56.32	2.04	0.41	22.25	2.21	1.21	14.89	1.21	0.38	23.18	**7.12** ^*^	**0.014**	35.87	0.92	0.63
	Sleep debt	56.48			22.37			14.37			26.06			36.57		
	Often insomnia	56.97			22.44			14.38			26.46			35.75		
Night snack	None	56.22	1.63	0.87	22.35	1.85	0.32	14.77	0.58	0.65	23.46	**6.45** ^*^	**0.034**	35.57	1.43	0.80
	1–3 time/week	56.46			22.19			14.20			25.95			36.02		
	≥4 time/week	57.10			22.52			14.69			26.27			36.59		

^*^P < 0.05, ^**^P < 0.01,^***^P < 0.001. The effects of age, Conscious health status, daily exercise time and daily Internet time on body shape indexes were analyzed by one–way ANOVA (the statistic is F value); The effects of leisure community participation, regular exercise habits, sleep habits and night snack habits on body shape indexes were analyzed by R × C contingency analysis (chi square x^2^ value).

### 3.4. Analysis of related factors affecting abdominal obesity of college students

Table 5 data display:

- The age of female college students had a significant effect on their visceral fat area (F=6.23, P=0.001 < 0.05), but had no effect on visceral fat grade, abdominal obesity rate (waist circumference ratio), visceral fat weight (kg) and subcutaneous fat weight (kg): conscious health status, daily exercise time and daily Internet time had no effect on the indexes of abdominal obesity.
- Regular exercise habits had a significant effect on visceral fat area (cm^2^) and visceral fat grade of female college students (x^2^ = 6.59*, P* = 0.0 7 < 0.05; x^2^ = 9.47, *P* = 0.033 < 0.05). It shows that the area of visceral fat is significantly smaller and the grade of visceral fat is significantly lower in those who have exercise habits: the sleep behavior of college students had a significant effect on their visceral fat area (cm^2^) and visceral fat grade (x^2^ = 26.11, *P* = 0.000 < 0.001; x^2^ = 14.3, *P* = 0.046 < 0.05). It showed that the better the sleep behavior, the smaller the visceral fat area and the lower the visceral fat grade.

**Table 5:** Statistical table of the influence of College Students' demographic variables on their abdominal obesity.

		**Visceral fat area (cm** ^**2**^ **)**			**Visceral fat grade**			**Abdominal obesity rate**			**Visceral fat weight (KG)**			**Subcutaneous fat weight (KG)**		
	**M**	**X** ^2^ **/F**	***P***	**M**	**X** ^2^ **/F**	***P***	**M**	**X** ^2^ **/F**	***P***	**M**	**X** ^2^ **/F**	***P***	**M**	**X** ^2^ **/F**	***P***
Age (years)	11.09	**6.23** ^*^	0.001	5.22	**3.49** ^*^	0.041	0.76	0.55	0.59	1.58	0.08	0.55	12.92	0.61	0.54
Conscious health status	11.09	0.51	0.48	5.22	1.34	0.36	0.76	0.28	0.73	1.58	−0.13	0.34	12.92	−0.13	0.33
Exercise time/day	11.09	0.65	0.81	5.22	0.26	0.54	0.76	1.26	0.25	1.58	−0.06	0.83	12.92	−0.05	0.72
Online time/day	11.09	0.78	0.43	5.22	0.59	0.47	0.76	0.08	0.81	1.58	−0.05	0.58	12.92	−0.08	0.48
Leisure participation	12.25	1.37	0.59	6.58	1.76	0.67	0.79	0.29	0.91	1.73	−0.25	0.80	14.11	−0.65	0.52
Regular exercise habits	13.69	**6.59** ^*^	0.037	8.11	**9.47** ^*^	0.033	0.81	1.32	0.31	1.68	1.48	0.14	14.88	0.80	0.43
Sleep	Enough sleep	9.52	**26.11** ^*^	**0.000**	3.48	**14.3** ^*^	0.046	0.73	2.68	0.61	1.46	1.48	0.14	12.12	1.02	0.34
	Sleep debt	11.08			5.20			0.76			1.63			13.32		
	Often insomnia	12.68			6.99			0.78			1.66			13.31		
Night snack	None	8.95	2.47	0.16	5.33	2.45	0.96	0.75	0.89	0.19	1.48	0.41	0.67	12.00	0.74	0.72
	1–3 time/week	11.20			4.52			0.75			1.62			13.91		
	≥4 time/week	13.13			5.81			0.79			1.63			13.84		

^*^P < 0.05, ^**^P < 0.01. The effects of age, Conscious health status, daily exercise time and daily Internet time on abdominal obesity were analyzed by one–way ANOVA (the statistic is F value); The effects of leisure community participation, regular exercise habits, sleep habits and night snack habits on body shape indexes were analyzed by R × C contingency analysis (chi square x^2^ value). Bold^*^ means statistically significant, with no other meaning.

## 4. Discussion

This study found that among the dimensions of health promoting lifestyle of female college students, self realization score was the highest, sport participation was the worst, followed by nutritional behavior. Wenwen et al. (20) conducted a survey of the current situation of health promoting lifestyle of nursing college students, which showed that the score of self realization was the highest, but the score of interpersonal support was the worst, followed by nutritional behavior. Fengcheng et al. (21) investigated the health promoting lifestyle of college students and found that interpersonal support scored the highest and sport or health responsibility scored the lowest. Lingyan et al. (22) investigated the health promoting lifestyle of college students with hypertension in Shanghai and found that the scores of interpersonal relationship were the highest and the score of sport behavior and health responsibility was the lowest. The research results of Guanghui et al. (23) show that the total score of healthy lifestyle of medical students is higher than that of other majors, so they believe that the healthy lifestyle of medical students is more ideal (24), but some scholars have reached the opposite conclusion (25). It can be seen that the findings of this study are basically consistent with the results of previous scholars, indicating that at present, Chinese college students may pay more attention to self realization in their health promotion lifestyle, and ignore sports participation, nutrition and health responsibility, indicating that college students still have a lot of room to improve their regular sports behavior and health care responsibility.

This study found that college students' self-health status was significantly positively correlated with their total score of self realization, sport participation, nutritional behavior and health promotion, which was also consistent with the research results of domestic scholar Guanghui (23). College students' self-health status can predict college students' healthy lifestyle, that is, the higher the score of self-health status, the better the healthy lifestyle. According to the cognitive behavior theory (26), positive cognition of health responsibility and health management will be embedded in individual health decision-making and practice as a continuous driving force, and further correct or improve their own health-related behaviors. In other words, college students can improve their exercise, nutrition and health responsible behavior by strengthening their awareness of self-health, so as to inhibit health dangerous behaviors such as smoking, drinking and Internet addiction (27). Jeong et al. (8) found that the health promoting lifestyle of college students is closely related to their age, major, leisure needs, students' personal characteristics and experience. The higher the grade, the lower the score of health promoting lifestyle, and the score of health promoting lifestyle of information college students is higher than that of other majors. This study did not analyze the differences in the scores of health promoting lifestyle among college students of different majors, but found that senior female college students' sports participation is poor, and the total score of health promoting lifestyle is the lowest, that is, the older they are, the worse their sports participation and overall health promoting behavior are. This finding confirms the views of Jeong et al. (8) and other scholars. Ting survey found that (28), college students' sports participation may decrease due to the increase of age, grade, school work and working time, that is, students' age has a significant negative correlation with sports dimensions, and it is speculated that the reason may be related to school work pressure.

This study found that those who did not participate in fixed leisure community activities and lack regular fitness habits had poor interpersonal support, inactive sport participation, improper nutrition and low total score of health promotion. With the increasing trend of adolescent obesity and maintaining a stationary posture for a long time, it is becoming more and more important to promote adolescent health, nutrition and exercise participation. The higher the pleasure value of participating in leisure activities, the better the health promoting lifestyle of college students (29, 30). Schwarz et al. (29) systematically reviewed the impact of leisure game on teenagers' health promoting lifestyle, indicating that teenagers' participation in leisure tourism has a significant predictive effect on their self realization, interpersonal support and stress coping. According to research of Guoxin and Xiaojuan (31), pleasant sport experience is conducive to the participation of associations. The more attention paid to lifestyle (such as interpersonal relationship and schoolwork orientation), the higher the demand for leisure and entertainment. The views of these scholars are very consistent with the findings of this study, that is, college students with fixed art and sport activity organizations or fixed leisure associations may affect college students' overall health promotion lifestyle, interpersonal support and sport performance.

This study found that college students' online hours affect college students' weight, which is likely to be related to students' lack of exercise due to too much online time. According to research of Guozai and Sanren (32), Internet addiction is one of the important risk factors for the damage of college students' health behavior. The longer you use the Internet, the lower your exercise participation and the worse your health quality of life. This study also found that regular exercise habits may affect the degree of visceral fat of college students. Relevant studies have reported that regular exercise is related to body fat content. The intervention of physical activities can effectively control the body fat rate and waist circumference of obese students (33, 34). Joseph et al. (35) found that the weight, body fat weight and BMI of male students in engineering colleges decreased after 12 aerobic exercise intervention training; Women who received 12 weeks of circular training can effectively reduce BMI, body fat rate, increase muscle mass, and significantly improve sleep quality (36). Rosa et al. (37) reviewed the improvement of health-related variables by independent physical activity intervention and reducing sedentary behavior, which showed that the results of the two interventions were similar, both had a significant degradation effect on the body fat percentage and body fat weight of various obese people and overweight people, and there was a significant upward trend in physical index, which could effectively improve body composition and cardiopulmonary fitness.

This study found that female college students' sleep behavior and regular exercise habits have a significant impact on their visceral fat area and visceral fat level, which is the same as that found in Theorell-Haglow et al. (38), that is, sleep habits may affect college students' visceral fat area. Krittanawong et al. (39) found that shorter sleep time is a risk factor for cardiovascular disease and increased mortality. Sleep time less than seven < 7 h (short sleep) and > 9 hohurs (long sleep) will increase the risk of cardiovascular disease death, especially in the Asian population and the elderly. Wheaton et al. (40) found that the daily sleep time of middle school students was negatively correlated with waist circumference, abdominal diameter and BMI. At the same time, less sleep time leads to reduced leptin secretion, increased brain intestinal hormone secretion, and increased appetite, all of which lead to obesity (41, 42).

## 5. Conclusions and suggestions

### 5.1. Conclusions

- Female college students pay more attention to self-realization in health promotion lifestyle, but ignore sports participation, nutrition and health responsibility. However, college students' self-health statuses can predict their healthy lifestyles, so college students can enhance their exercise, nutrition and health responsibility behavior by strengthening their awareness of self-health.
- Female college students' participation in leisure activities and regular fitness habits can significantly predict their self-realization, interpersonal support and stress coping. Regular exercise habits may affect the degree of visceral fat, while Internet hours negatively affect college students' weight and health quality of life.
- The age of female college students has a significant impact on their visceral fat area. Regular exercise habits affect their visceral fat area and visceral fat level, while the better their sleep behavior, the lower their visceral fat area and visceral fat level.

### 5.2. Suggestions

- This study adopts a cross-sectional research design and lacks long-term follow-up investigation. It may not be possible to make causal inference on the related factors affecting health promoting lifestyle and body composition (state and abdominal obesity). It suggests that future research can be tracked sustainably.
- This study only takes the students of a comprehensive university in Central China as the research object, so the research results cannot be inferred to the female college students in the whole central region. There may be regional differences between different regions. It is suggested that future research can increase the scope of sample size and expand to the comparison between different schools, so as to improve the representativeness and value of the research.

## Data availability statement

The original contributions presented in the study are included in the article/supplementary material, further inquiries can be directed to the corresponding authors.

## Ethics statement

This study was approved by the Ethics Committee of School of Physical Education, Southwest Medical University (Approval No.: swmu-ty2022002). The patients/participants provided their written informed consent to participate in this study.

## Author contributions

XL contributed to the conception or design of the paper and drafted the manuscript. HL contributed to the acquisition, analysis, or interpretation of data for the work. XL and HL have read and agreed to the published version of the manuscript. All authors contributed to the article and approved the submitted version.
